# Why We Belong - Exploring Membership of Healthcare Professionals in an Intensive Care Virtual Community Via Online Focus Groups: Rationale and Protocol

**DOI:** 10.2196/resprot.5323

**Published:** 2016-06-13

**Authors:** Kaye Rolls, Margaret Hansen, Debra Jackson, Doug Elliott

**Affiliations:** ^1^ Agency for Clinical Innovation Intensive Care Coordination and Monitoring Unit Chatswood Australia; ^2^ University of Technology Sydney Faculty of Health Sydney Australia; ^3^ University of Syndey Sydney Nursing School Sydney Australia; ^4^ University of San Francisco School of Nursing and Health Professions San Francisco, CA United States; ^5^ Oxford Brookes University Faculty of Health and Life Sciences Oxford United Kingdom; ^6^ NHS Foundation Trust Oxford University Hospitals Foundation Trust Oxford United Kingdom; ^7^ University of New England School of Health Armidale Australia

**Keywords:** focus groups, virtual communities, social media, qualitative methods, clinicians, intensive care

## Abstract

**Background:**

Many current challenges of evidence-based practice are related to ineffective social networks among health care professionals. Opportunities exist for multidisciplinary virtual communities to transcend professional and organizational boundaries and facilitate important knowledge transfer. Although health care professionals have been using the Internet to form virtual communities for many years, little is known regarding “why” they join, as most research has focused on the perspective of “posters,” who form a minority of members.

**Objective:**

Our aim was to develop a comprehensive understanding of why health care professionals belong to a virtual community (VC).

**Methods:**

A qualitative approach will be used to explore why health care professionals belong to an intensive care practice-based VC, established since 2003. Three asynchronous online focus groups will be convened using a closed secure discussion forum. Participants will be recruited directly by sending emails to the VC and a Google form used to collect consent and participant demographics. Participants will be stratified by their online posting behaviors between September 1, 2012, and August 31, 2014: (1) more than 5 posts, (2) 1-5 posts, or (3) no posts. A question guide will be used to guide participant discussion. A moderation approach based on the principles of focus group method and e-moderation has been developed. The main source of data will be discussion threads, supported by a research diary and field notes. Data analysis will be undertaken using a thematic approach and framed by the Diffusion of Innovation theory. NVivo software will be used to support analyses.

**Results:**

At the time of writing, 29 participants agreed to participate (Focus Group 1: n=4; Focus Group 2: n=16; Focus Group 3: n=9) and data collection was complete.

**Conclusions:**

This study will contribute to a growing body of research on the use of social media in professional health care settings. Specifically, we hope results will demonstrate an enhancement of health care professionals’ social networks and how VCs may improve knowledge distribution and patient care outcomes. Additionally, the study will contribute to research methods development in this area by detailing approaches to understand the effectiveness of online focus groups as a data collection method for qualitative research methods.

## Introduction

Contemporary organizational [1] and learning theories [2] that highlight learning and behavior are influenced by social networks [3,4]. Many of the current challenges of evidence-based practice [3] are related to the ineffective social networks [5] created by professional and organizational boundaries [6,7]. While there is significant potential within multidisciplinary virtual communities (VC) to facilitate the transfer of research and best practice [6,8] and support the professional development of clinicians [9], at this time we know little of why health care professionals (HCPs) join or how they use a VC. The purpose of this paper is to present a research protocol for a study that aims to develop an understanding of why HCPs join a practice-based VC and how they use it.

### Influence of Local Social Networks on Clinical Practice

**Figure 1:**
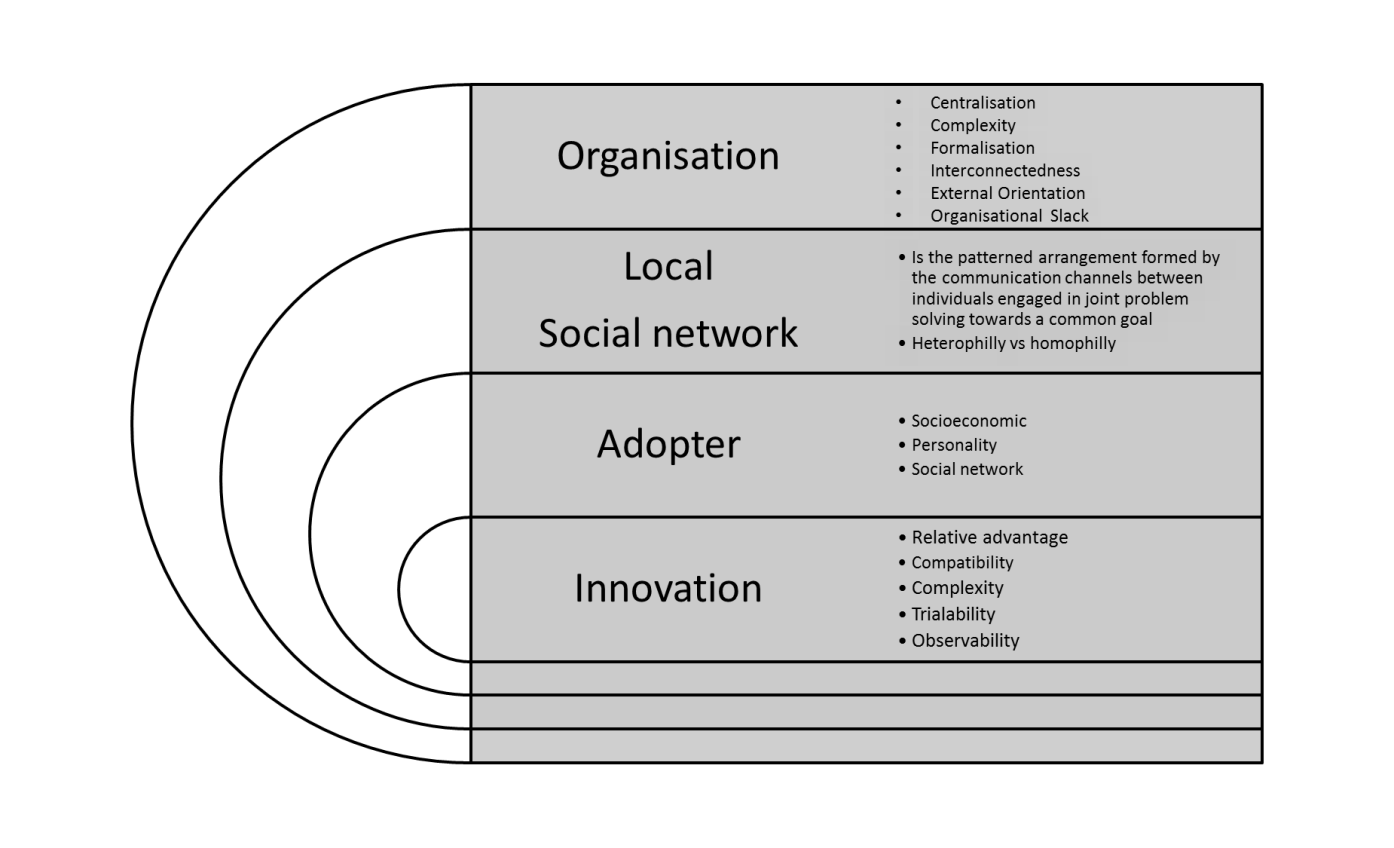
Diffusion of Innovation 5, 13.

For 30 years, evidence-based practices have been viewed as the gold standard. However, significant clinical practice variation and evidence-practice gaps persist [10-12]. According to Rogers’ Diffusion of Innovation theory [13], the adoption of an innovation, such as new practices, research, or technologies, is mediated by characteristics of the innovation, an individual’s adoption style and their social network, the broader social context, and time (see Figure 1 and Multimedia Appendix 1). Access to novel information requires a heterogeneous social network (where members may not have similar values and characteristics) where communication channels cross organizational and/or professional boundaries [14]. Furthermore, trials and final adoption decisions are strongly influenced by opinion leaders and peers [13,15-18]. However, current research suggests that the preferred information sources of many clinicians are a function of perceived credibility and ease of access [19-21] and that professional networks shape and limit clinical behaviors [4]. If clinicians do not have communication channels beyond local networks, they will not have access to novel knowledge and may be under the illusion that local practices reflect the majority [22]. Social media have the potential to improve HCP social networks by creating multidisciplinary VCs that facilitate knowledge exchange regardless of geography or time [6-8].

### Virtual Community Use by Health Care Professionals

Health care professionals have been using VCs since the early 1990s with long-term success stories including (1) Critical care mailing list launched in 1994 [23], (2) NurseNet founded in 1993 [24], and (3) MEDLIB started 1991 [25]. These VCs were created using early social media technologies including listserv and discussion forums [26]. The advent of Web 2.0 and the newer technologies of social networking and microblogging platforms have expanded the possibilities of professional networking and perhaps virtual communities [8]. While reasons for establishing a discrete VC vary, the most common motivation was to create a professional forum where relevant professional and academic issues may be discussed and knowledge shared [27-39]. Unfortunately at this time, membership of many health care VCs is often homophilic (ie, the tendency to associate with individuals who share similar values and characteristics [13]) where members are commonly from a single health care discipline and usually work in a specific clinical specialty area [25,31,37,40-42]. Additionally there are limited population-based data describing how different types of HCPs are using the variety of social media platforms. In 2011, as few as 1.7% of emergency physicians were using Twitter [43] and 13.4% of Korean emergency physicians were using a Facebook page [44], whereas up to 20% of intensive care nurses [14], occupational health practitioners [45], or nurse practitioners [34] were using a professional listserv established for their use. These differences in uptake, and that each technology will be seen as an innovation, indicate a possible mediating factor related to the social medium itself [46] as well as the influence of peers [14,47].

The most common online activities undertaken by VC members are the solicitation and supply of experiential domain-specific knowledge [30,45,48]; however, 60-89% of members rarely post online [42,45,48,49]. A limited number of studies suggest that HCPs view VCs as valuable knowledge portals, enabling members to remain clinically current [50] with relevant and quality information [49,51], develop workplace resources [41], and benchmark practice [41,50]. This suggests HCPs use VCs to establish virtual professional networks [13] to enhance access to colleagues and best practice knowledge.

A reliance on readily available data and use of online observation has limited our understanding of how or why HCP use social media because this gathers data on a limited number of members. Given this, what motivates HCPs to join a VC, and what do they value that influences them to remain members? The absorption and diffusion of knowledge or innovation into and around an organization is the role of boundary spanners (eg, nursing unit managers or project officers) [52] and knowledge brokers (eg, nurses in education or advanced practice roles) [53]. Do these individuals see membership as part of personal professional development or as a tool for their substantive position, as preliminary data suggest [41]? Additionally, understanding these phenomena will assist health care leaders in understanding how to develop VC to optimally leverage social media to improve knowledge diffusion and patient care.

### Online Focus Groups

Focus groups are used by researchers to gather qualitative data on specific group experiences by capitalizing on group dynamics to synergistically develop a deeper, richer understanding of a phenomenon of interest [54,55]. Moreover, the collective conversation between participants facilitates the gathering of individual and group voices, which may uncover an understanding not available via other data collection modes (eg, surveys or interviews) and democratizes the research by decentering the researcher [54]. A moderator guides participants through a discussion commonly using a guide based on the core research questions and objectives and evolves as data emerge [55]. While face-to-face focus groups are acknowledged as a strong method for gathering qualitative data [55], there can be significant logistical challenges, such as convening the focus group on a specific date and time and at a location that facilitates maximal participation. Online or virtual focus groups are becoming more common as they enable participation of geographically distributed and time-poor individuals and are less expensive to conduct [55,56]. Online focus groups have been used to examine a diverse range of health-related questions, with considerable variation in methods used across studies (see Table 1). While the term “virtual focus group” is more commonly used, we use the term “online focus group” to avoid confusion with the term “virtual community.”

**Table 1:** Use of virtual focus groups in health^a^.

Author, year, country	Aim	Focus group + participants	Running the virtual focus group (VFG)	Data analysis
Alonzo, 2009, USA [61]	What motivates associate degree and diploma-prepared RN to pursue a baccalaureate degree through an RN-to-BSN program	4 VFG (2-6 participants); nurses; 2 weeks	Asynchronous using discussion forum and a question guide (11)	Inductive content analysis
Synnot, 2014, Australia [62]	Compare face-to-face and VFG for people with multiple sclerosis & relatives regarding needs, experiences, preferences, and values when integrating evidence-based health information into their decision making about the management of their health	4 face-to-face (27participants); 1 VFG (33 participants) over 2 months	Asynchronous using discussion forum; 10-question guide	Thematic analysis
Hanson, 2011, USA [63]	To explore fieldwork educator motivations for working with students and the kind of support needed from the academic institution (occupational therapists enrolled in master’s program)	2 VFG based on stratification to pediatric & adult practice settings (10 participants); over 2 weeks; credit incentives for participation	Asynchronous using discussion forum; all questions posted at start with instructions for students to respond to each question plus 2 peer responses	Content analysis
Tates, 2009, Netherlands [64]	Determine what constitutes good quality of communication with a diagnosis of childhood cancer, in terms of participation and role delineation from their point of view	3 VFG grouped by type (7 current patients, 11 parents of these patients; 18 survivors)	Asynchronous using discussion forum; daily questions over 1 week	Not described
Harmsen, 2013, Holland [65]	Gain insight into factors that influence parents to not vaccinate their children	8 VFG; 5 non-vaccinators (n=39; 7-9); 3 partial (n=21; 7 each); running over 5 days	Asynchronous using discussion forum; predetermined topics introduced daily with open questions; anonymous	Thematic analysis
Murray, 2001, International [66]	To test method and gather data to inform interviews; part of a mixed methods study to gather data & test method	2 VFG ‒ Educators and listserv experts (N not provided); 4 weeks	Asynchronous using listserv	Not explained
Adler, 2002, USA [67]	VFG as mode of data collection; lived experience of women confined to best rest because at risk of preterm labor; value of VFG as peer support	1 FG (7); 4 weeks	Asynchronous using listserv; Question guide – 6 (semistructured, open ended)	Content analysis for thematic coding
Kenny, 2005, Australia [51]	Whether active engagement and group interaction could be captured in an online environment in an EN conversion program	1 FG with census sample	Asynchronous using Web CT starting with one question; ran for 2 months	Thematic analysis
Pechak, 2002, USA [68]	Develop recommendations for implantation of ICE in physical therapist education to promote ethical practice	1 VFG (5 participants); followed by 3 delphi rounds (19 participants)	Synchronous using Blackboard; anonymous; highly structured feedback on predetermined script	Not described
Levine, 2011, USA [69]	Involve youth of color in design of programmatic content and formats for an Internet intervention for sex education	4 synchronous FG (7,5,4,2 participants); 1 asynchronous (18 participants)	Synchronous using chat room (4 by 1 hr); switched to asynchronous due to low numbers – 7 days with daily questions (9 in total)	Not described
Brubaker, 2012, USA [70]	Gather information about women’s knowledge and attitudes regarding research participation	2 FG grouped by research-experience or research-naive (12 in total); study protocol also include 14 face-to-face FG	Synchronous using semistructured discussion guides	
Tuttas, 2014, USA [71]	Capture travel nurses’ perceptions of boarding experiences	4 FG (2-5 participants); registered nurses	Synchronous using Web conferencing and a question guide (5 questions); over 45-60 minutes	Qualitative content analysis

^a^BSN‒baccalaureate science nursing; EN‒enrolled nurse; FG‒focus group; ICE ‒international clinical education; RN‒registered nurse; VFG‒virtual focus group.

Two modes of online focus groups are possible: synchronous and asynchronous. The synchronous mode closely matches face-to-face groups where participants meet in real time using chat rooms or discussion boards. While this mode may promote a more dynamic discussion with high levels of feedback, an individual’s typing speed, connection bandwidth, and thought speed may impact users’ ability to effectively participate [55]. Asynchronous groups have been conducted using either listserv or discussion forum technology, providing participants with time to consider their posts or responses, and enable posting at a time of their convenience. Other advantages of the asynchronous mode include immediate creation of a threaded discussion facilitating review by members as well as data collection and analysis [55,57]. Study credibility is enhanced [58] by participant-controlled, real-time data collection. While the asynchronous mode may facilitate the development of more reflexive answers [56], large participant numbers may create two methodological issues: (1) the quality of interaction, and therefore data, may be limited because the volume of posts is off-putting and/or too high for participants to review properly, and (2) moderation is more challenging. A high volume of data also may make data analysis more difficult.

As noted above, considerable variation exists regarding how researchers structure online focus groups (see [55]. In addition, most VC members do not actively post online and the decision to post is complex [59,60]. Focus group participants may be more inclined to disclose their experiences and opinions where they feel they share values and beliefs with other group members and there is no group hierarchy [61]. This homogeneity along with efficient moderation can lead to effective group interactions resulting in quality data [55]. The ideal moderator understands both the context of the research and the cultural world of participants [62,63]. However, effective online moderation requires additional skills and interventions that socialize participants to the online space and encourages posting [64]. Two other important considerations are that the platform chosen is user-friendly (ie, easy to access and use and esthetically pleasing) [65] and the posts are confidential [66].

A key component of a focus group is the discussion guide that frames and focuses discussions and ensures collection of rich in-depth data [55,62]. Questions should reflect the study questions and funnel discussions through introductory, transition, and key questions to ensure consistent data where multiple groups are used and aid data analysis [67]. Introductory questions encourage participation and provide participants and researchers with an understanding of individual perspectives [55]. These are similar to activities undertaken as part of an e-moderation process to support effective online learning, including establishing an effective group, introduction of the research phenomena, and induction of participants to the online environment [64].

### Focus Groups and Virtual Communities

While focus groups are frequently used to collect data for qualitative or mixed-methods studies, only two studies [31,68] were identified that examined HCP experiences of virtual communities or computer-mediated communication. In a mixed-methods study exploring how and why occupational therapists used a virtual community of practice (VCoP), two face-to-face focus groups (stratified by use or not of the VCoP) were used to develop a survey instrument [31]. In earlier work [68], two online asynchronous focus groups were convened using listserv technology to explore current practice and future potential of text-based computer-mediated communication as a mechanism for qualified nurses to meet their formal and informal continuing professional development needs. In this latter study, questions were introduced at the beginning of the focus group, with the author later reflecting that this was overwhelming for some participants [69]. While listserv technology is the most straightforward and accessible of all VC platforms, it may not result in a chronologically ordered discussion thread. This may be difficult for both the participants and moderator to follow the discussion, especially those with multiple posts, and could therefore limit interaction and conversation development with probable negative effects on data. Data analysis is also more complicated because of difficulties in understanding the chronology and/or evolution of a discussion.

The aim of this study is to explore why HCPs belong to an intensive care practice-based VC. The main objectives are to (1) understand why members join and remain members, (2) identify what purpose the VC serves in in their professional lives, (3) identify how a member uses the VC, and (4) identify how they have used the knowledge or resources shared on the VC.

## Methods

A qualitative approach will be used to collect data using three asynchronous online focus groups, with participants allocated to a group based on their posting behaviors in the past 2 years. The study is framed by the Diffusion of Innovations theory [13]. A summary of the protocol is provided inFigure 2.

**Figure 2:**
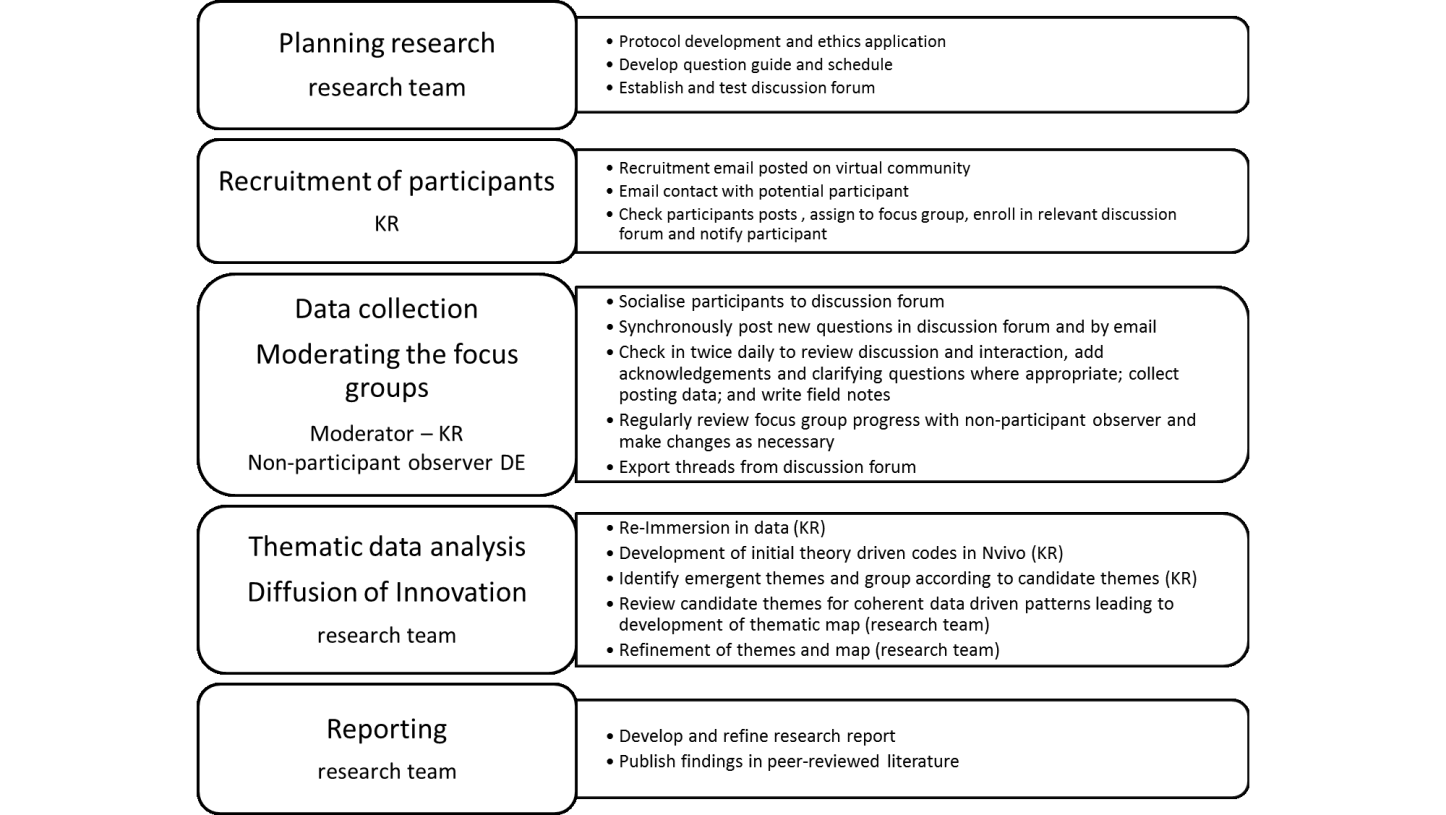
Study protocol summary.

### Ethics

Approval has been granted by a University Human Research Ethics Committee (UTS HREC REF NO. 2014000378). Participant confidentially will be ensured using two measures: (1) a group rule, covering non-disclosure of participant names or sharing the content of posts, will be developed and participants will be asked to agree to it on participant registration, and (2) focus groups will be convened within a secure website using a closed, password-protected discussion forum with any social media sharing function disabled. These layers are designed to protect participant anonymity and prevent forum posts from being searchable via the Web [66].

### Setting

The VC is a professional listserv established in 2003 by an Australian state health department to reduce the sense of professional isolation and improve knowledge distribution between the 43 intensive care units [51]. By mid-2014, there were more than 1700 members from more than 225 health care facilities, universities, and industry partners, spread throughout several countries with most being Australian intensive care clinicians and nursing being the largest professional group. Analysis of the social network of the VC suggests that it is highly valued by members because the majority of HCP who join choose to remain members for extended periods and recommend the VC to colleagues [14]. The VC would be classified as a VC with an interdisciplinary culture and stable membership, a medium geographic distribution, and an open and voluntary enrollment [70].

### Participants and Sample

A purposive sampling method will be used to recruit between 24 and 36 participants for the three focus groups. The sample size is based on recruiting 8-12 participants per group, which is the current recommendation for both traditional [55] and online [65] focus groups. Members of the VC will be invited to participate via a recruitment email posted to the VC, providing all participant information, and an invitation to contact the research team for further information, and a link to the online recruitment form (Google forms). The online recruitment form will include participant information, consent, participant demographics, and a short survey covering group rules (see Multimedia Appendix 2). Once a potential participant has completed the online registration and consent, their posting behavior will be checked, they will be assigned to a focus group, and they will be notified of the details regarding this focus group.

To develop an understanding of a range of member types, we chose to undertake three focus groups based the online activity of a VC member. Participants will be purposely assigned to a focus group based on their posting activities on the VC in the last 2 years (onlist posting) (September 1, 2012, to August 31, 2014): (1) more than five times, (2) five times or less, and (3) not posted. We will not cap the number of participants as dropouts or inabilities to participate have been identified as limitations by previous researchers [71,72]. The only exclusion criteria will be non-availability over the 3-week time frame for each focus group.

Recruitment challenges are anticipated. As the majority of VC members do not post, this reduces the number of potential candidates for the posting groups particularly for Groups 1 and 2. A review of 12 months of activity identified at least 25 members eligible for focus Groups 1 and 2. While there are a high number of potential members for the non-posting group, these members are reluctant to post for a variety of reasons, especially about how their contribution might be received by members of the virtual community. We hope that by convening focus groups where the shared characteristic is posting, behaviors will create an online space where individual members feel comfortable and confident that their contributions will be met in a positive and supportive environment [56].

### Moderation

The approach to moderation of the focus groups is based on principles from moderating traditional focus groups [55] and facilitating learning online or e-moderating [62-64]. The first author will be the moderator and is an experienced intensive care nurse and was the previous moderator of the VC. Author 4 will be a non-participant observer. To facilitate access to and understanding of the focus group platform, a “how-to” guide has been developed as an important component of providing access and motivation to post in an online forum [64].

### Running the Online Focus Groups

The focus groups will be run over 3 weeks using a closed discussion forum (IPBoard version 3, Invision, Powerboard) hosted on a secure jurisdictional health department website. The focus groups will be held consecutively, which will allow for refinement of the question guide based on data from a previous group [73]. This approach was developed to enable optimal participation and interaction, safeguarding participant confidentiality, facilitating moderation and effective data collection. The host site was chosen as it was accessible and useable across fixed and mobile technologies. A discussion forum was chosen for a number of reasons. Discussion forums are asynchronous and create a chronological electronic record where participants will be able to review what has been posted, have time to consider and formulate a response, and then post at a time convenient to them [57]. This should promote a more egalitarian focus group as all participants will have the opportunity to provide input. This enhances participant control and may encourage more detailed and reflective answers, and thus potentially richer data [55]. As the discussion moves forward, a record is created providing participants with chronological discussion points and data collection is facilitated through the development of discussion threads.

### Question Guide and Discussion Schedule

A question guide (see Table 2) was developed with questions based on the research questions and the theoretical framework of diffusion of innovations [13]. A schedule will be developed with new questions posted every 2-3 days depending on how the discussion is developing. To facilitate visibility of the study and new questions, an email using a standardized subject heading will be sent to participants alerting them to new content. The moderator will access the forum at least twice each day for promoting interaction (eg, reviewing posts, answering questions, or adding additional questions to clarify participants’ views) [71,72], regularly thank and encourage participants, and re-inforce the value of posting [64].

**Table 2:** Question guide.

Type of question	Questions	Possible aspect of diffusion of innovation^a,b^
Introductory question	Please introduce yourself and tell the group about your professional role and experience.	
Transition question	You were invited to this focus group because you are a member of ICUConnect. Could you explain what prompted you to join?	Type of adopter; homophily; influence of peers
	Do you use any other social media or online communities for professional networking and development?	Type of adopter; external orientation; interconnectedness; Innovation characteristics of social media
Key question	What do you value most about ICUConnect?	Access to colleagues (homophily), external orientation; interconnectedness; Innovation characteristics of social media
	What are the least valuable aspects of ICUConnect?	Innovation characteristics of social media
	What advantages or disadvantages does ICUConnect have over other social media?	See above
	Current research indicates that there are active users of virtual communities (individuals who post) and passive users (individuals who mainly read &/or share). How would you describe how you use ICUConnect?	Type of innovator: role of individual in local social network
	Do you share ICUConnect posts with other professional colleagues?	Role of individual in local social network; external orientation
	Is there a post in the past 3 months that has been of high relevance to you?	Knowledge (innovation) on IC-VC is credible
	Have you been able to use any posts from the last 6 months of discussions?	As above
Concluding question	Are there any other important aspects of ICUConnect that we have not discussed?	As above

^a^[5,13].

^b^See Figure 1 and Multimedia Appendix 1.

### Data Analyses

Study data will include (1) demographic data describing participant characteristics, (2) categorical data describing discussion forum participation, (3) discussion threads documenting focus group discussion, and (4) field notes. Discussion threads will be extracted from the Forums using NCapture (QRS International). NVivo (QRS International) will be used to manage data analyses. A research diary and field notes will be maintained to support analyses. Initial analyses will be conducted by KR, supported by scheduled reviews with the research team to evaluate progress and reach consensus regarding themes and other interpretations. Analysis of the discussion threads will be undertaken using a thematic approach [74], framed by the diffusion of innovation [13]. Thematic analysis is a 6-phase process allowing the researcher to systematically identify, analyze, and report patterns found in qualitative data [74]. During Phase 1, KR will be immersed in the data through active reading of discussion threads and looking for meanings or patterns. This familiarization will commence during data collection because of the dual role of researcher-moderator. DE also will be familiar with the data in his role as researcher-participant observer. During Phase 2, the initial codes will be generated; these codes will be theory driven and will represent the most basic element of the raw data that is meaningful. Additionally, code descriptors will be developed to ensure systematic coding. In Phase 3, we will look for themes by grouping codes into candidate themes. In Phase 4, we will refine this list of themes by reviewing the coded extracts and looking for a coherent pattern within each theme and ensure there is sufficient data to support it. Once we have achieved this, we will move on to developing the thematic map, which reflects how well the themes represent the data as a whole. Re-reading the whole dataset is essential, and some recoding may be required at this point. During Phase 5, we will define and refine the themes by identifying the essence of the theme and determining which aspect of the data it captures. This involves developing a detailed analysis of each theme and its associated subthemes. In the last phase, we will provide a written report of our analyses.

Diffusion of innovation [13] was chosen as the theoretical lens because the research team felt it was a better match for both the broad problem of inadequate social networks limiting knowledge diffusion in health care and the current gaps in the literature. While other behavioral models, including theory of reasoned action [75], theory of planned behavior [75], and technology acceptance model [46], have been used and produced important insights, they are focused on an individual’s behavior.

### Study Quality

Rigor in qualitative research is a contentious area [58,76,77]. The preferred terms of “trustworthiness” or “confirmability” reflect accuracy and comprehensiveness in how data were collected, analyzed, and reported. For this study, several strategies will be used. Credibility of data will be enhanced as participants have direct control over their contributions that will be recorded in real time, and by use of NVivo software as the major study file repository for the research diary, field notes, and data, thus establishing a clear audit trail [78]. A “thick” description of the research context will be provided by describing the participants (using the recruitment survey), virtual community, and research process (p. 69 [58]). Auditability will be supported by field notes, recording impressions arising from focus groups, and NVivo to manage data analyses. Data credibility will be enhanced by presenting preliminary themes to participants for early review (member checking) [56].

Field notes record what the researcher experiences during data collection and includes (1) both a description of and reflection on what occurred, (2) a reflective journal that includes personal thoughts and feelings, and (3) any insights, judgments, and interpretations made in the field [76,78]. Field notes will facilitate both data collection (eg, aid in development of elaboration and clarification questions) and analysis (eg, through the development of preliminary themes).

### Researcher Bias and Relationship With Participants

The potential for bias in qualitative research may be significant when the research team fails to understand and then manage their assumptions and biases. In addition, where there is an unequal or prior relationship between the research team and participants, data collected may not reflect the reality of participant experience. In this study, KR was a long-term moderator of the VC and DE is a member. However, the other authors are not members or associated with the VC. To manage any potential for bias during data collection and analyses, a number of procedures will be implemented: (1) KR withdrew from the moderator role several months prior to VC members’ being aware of the research (all stages of the study), (2) to minimize coercion in all communications, KR will describe participants in a passive research guise and will not make any direct communications with individual members, (3) KR will undergo a bracketing process prior to the first focus group, outlining the researcher position by documenting any assumptions and therefore identifying potential sources of bias [79,80], and forming part of the research diary, (4) assumptions will be revisited during data analyses, (5) during focus group moderation, the roles as researcher (KR) and non-participant observer (DE) will be explicitly described, (6) to minimize bias and enhance credibility, all researchers will be responsible for data analysis, and (7) member checking will be undertaken by posting preliminary results in the discussion forums for participants to provide feedback.

## Results

At the time of writing, 29 VC community members have been recruited and the focus groups were conducted October to December 2014 with these participants. Table 3 shows focus group recruitment outcomes and professional roles of participants. There was mixed participation across the focus groups (3, 9, and 7 respectively), which may create challenges for data analyses.

**Table 3:** Focus group recruitment outcomes.

Type of member	Focus group 1: Frequent posters (>5)	Focus group 2: Low posters (1-5)	Focus group 3: Non posters	Total
Clinical nurse-internal^a^		4	2	6
Clinical nurse-external^b^		1	1	2
Knowledge broker nurse^c^	3	4	2	9
Clinical unit manager^d^	1	2	1	4
Academic nurse^e^		4	1	5
Physiotherapist			1	1
Physician		1		1
Bureaucrat^f^			1	1
Total	4	16	9	29
Post range	6-19	1-4 (mode 1; median 1)		

^a^Clinical nurse‒internal provides clinical services within a clinical unit.

^b^Clinical nurse‒external provides clinical services across multiple clinical unit.

^c^Knowledge broker job role could include advanced practice, education, research, or practice development.

^d^Clinical unit manager manages a defined ward or clinical area.

^e^Academic nurse is employed by a tertiary education institution.

^f^Bureaucrat is employed in a non-clinical or managerial role in health service.

## Discussion

### Principal Considerations

Like the rest of the community, HCPs are adopting social media platforms, although uptake varies considerably [14,34,43,44]. Despite positive attitudes towards social media, this has not translated to significant professional use [81]. There are some data suggesting this is influenced by individual characteristics [46], peers [46,82], and perceptions of the platform as an innovation [46]. At this time, however, the research base on why or how HCPs use these communities or social media is limited because online observation reveals the perspective of a minority of VC members [25,27,32,42,45,49,83] and measurement [25,36,44,49,84-87] and sample [31,49,84,88-92] bias in surveys. It has been suggested that a comprehensive understanding of VCs requires a mixed-methods approach that includes a member survey, content analysis, and social network analysis [93]. Social network analysis has revealed that members have more complex reading than posting behaviors [39]; however, this will not reveal member motivations and will be limited to platforms where these data are available.

The aim of this study is therefore to develop a comprehensive understanding of why members belong to an intensive care practice-based virtual community for HCPs. This includes understanding why they join and remain members, identifying the purpose of the VC in their professional lives, and understanding how they use the VC, and what they do or how they use the knowledge or resources obtained. By using focus groups, we will be able to examine the experiences of all types of VC members, leading to a more complete understanding of why HCPs join and use social media. By using the diffusion of innovation as a theoretical lens, we also examine the phenomena from several perspectives including social media as an innovation, the VC as an IC-VC, VC member adopter type, and VC as a social network. We hope to show that VC membership enhances the professional (social) networks of HCPs and access to valuable knowledge, to improve clinical practice, and by extension patient outcomes.

There are a number of possible benefits arising from this study. The study will provide data about participation in this VC, particularly as a method to support evidence-based practice and professional development [8] and address patient care challenges [6]. As this VC is part of a jurisdictional health department initiative, the health system and broader community may benefit by demonstrating the viability and value of social media to improve the social networks of intensive clinicians and as a knowledge diffusion and adoption initiative. Findings may also allow the development of a survey instrument to gather data from a larger sample of VC members and on other VCs. This study will also contribute understanding on the efficacy of online focus groups as a data collection method for qualitative research methods.

### Strengths and Limitations

There are several strengths and limitations of this study. Two elements limit generalizability to the broader population of HCPs, namely the qualitative design using focus groups and the Australian intensive care setting. However, in the current literature, generalizability of surveys is hampered by sampling bias [31,49,84,88-92]. Our design leverages the advantages of online focus groups with learnings from virtual tertiary education [64] to facilitate participation by a broad range of members thus providing an extensive understanding of the experiences of all types of members, especially the non-posting majority. Our recruitment has been moderately successful in gaining adequate participants for the low and non-posting focus groups but not for the high-posting group [65]. This may limit the quality and quantity of data arising from this focus group.

### Conclusions

This study aims to contribute to the growing body of research on the use of social media; specifically we hope it will demonstrate this by enhancing access to social networks for HCPs. VCs may improve collegiality, data sharing, knowledge distribution, and ultimately patient care and health outcomes. Additionally, the study will contribute to qualitative research methods by evaluating the utility of online focus groups as a data collection approach.

## Appendix

Multimedia Appendix 1 Description of terms used in Diffusion of Innovations framework.

Multimedia Appendix 2 Online recruitment - demographics and group rules.

## Abbreviations

HCP: health care professionals
VC: virtual communities
VFG: virtual focus group
