# Supplementary material for: Why We Belong - Exploring Membership of Healthcare Professionals in an Intensive Care Virtual Community Via Online Focus Groups: Rationale and Protocol
Source: JMIR Res Protoc. 2016 Jun 13;5(2):e99. doi: 10.2196/resprot.5323 (PMC4923593; doi:10.2196/resprot.5323)
Supplement: Multimedia Appendix 1 [file resprot_v5i2e99_app1.pdf]

Multimedia appendix 1 Description of terms used in Diffusion of innovation framework

Aspect of Diffusion of innovation	Characteristic	Descriptor
Innovation [1]	Relative advantage	The degree to which an innovation is perceived to be an improvement on what it supersedes
	Complexity	The degree to which an innovation is perceived to be challenging to comprehend or use
	Compatibility	The degree to which the innovation is perceived to be congruent with values, experiences and needs of potential adopters
	Trialability	Whether an innovation can be trialled before making an adoption decision
	Observability	Whether the outcomes of adoption of an innovation are visible to potential adoptees
Individual – Adopter<br><br>Innovators – 2.5%<br>Early adopters – 13.5%<br>Early majority – 34%<br>Late majority – 34%<br>Laggards – 16%	Socioeconomic	Early adopters generally have more years of formal education, a higher social status and higher disposable income [1]
	Personality	Early adopters are better able to deal with the abstract, uncertainty and risk, have more empathy and are less dogmatic than later adopters [1]
	Personality	Early adopters are better able to deal with the abstract, uncertainty and risk, have more empathy and are less dogmatic than later adopters [1]
	Social network	Early adopters have larger and more varied communication channels (including mass media) which extend beyond local boundaries [1]
Social system [1]	Definition	Is the patterned arrangement formed by the communication channels between individuals engaged in joint problem solving towards a common goal
	Homophilly	The degree to which individuals who communicate are similar (that is share beliefs, values, socioeconomic status and education). A highly homophilous social system aids diffusion however new knowledge will be difficult to access as redundancy is reached quickly

Aspect of Diffusion of innovation	Characteristic	Descriptor
	Heterophilly	The degree to which individuals who communicate are different (that is share beliefs, values, socioeconomic status and education). A highly heterophillic social system will have greater access to novel knowledge but diffusion will be made difficult due to lack of individual trust
	Communication channels	Interpersonal communication channels are where information is exchanged between two individuals whereas mass media channels involve information exchanged from one to many. While mass media channels enable access to a broader range of information interpersonal channels, or peer-to-peer knowledge exchange are more influential on decisions to try and then finally adopt an innovation [1]
Organisation	Centralisation	degree to which power and control are concentrated in few individuals [1]
	Inter-connectedness	Degree to which separate units are in the organisation are linked by social networks [2]
	Organisational slack	degree to which uncommitted resources are available [1]
	External Orientation	Where organisational leaders have professional networks external to their workplace [3, 4]
	Formalisation	degree to which an organisation emphasises rules and regulation [1]
	Complexity	degree to which members possess a high level of knowledge and skill [1]
	Absorptive capacity	ability of an organisation to acquire, assimilate and exploit new knowledge for organisational advantage[5]
	Complexity	degree to which members possess a high level of knowledge and skill [1]

1. Rogers EM. Diffusion of Innovations. 5th ed. New York: Free Press; 2003. 978-0-7432-2209-9
2. Nieves JOsorio J. The role of social networks in knowledge creation. Knowledge Management Research and Practice 2012;**00**:1-161477–8238/12
3. Aarons GA, Hurlburt MHorwitz SM. Advancing a conceptual model of evidence-based practice implementation in public service sectors. Administration and Policy in Mental Health and Mental Health Services Research 2011;**38**(1):4-23.14 December 2010,(10.1007/s10488-010-0327-7.
4. Purcell RMcGrath F. The Search for External Knowledge. Electronic Journal of Knowledge Management 2013;**11**(2):158-167.
5. Ehin C. Hidden assets: harnessing the power of informal networks. London: Kluwer Academic Publishers; 2004.
